# Escape dynamics of active particles in multistable potentials

**DOI:** 10.1038/s41467-021-22647-6

**Published:** 2021-04-27

**Authors:** A. Militaru, M. Innerbichler, M. Frimmer, F. Tebbenjohanns, L. Novotny, C. Dellago

**Affiliations:** 1 Photonics Laboratory, ETH Zurich, Zurich, Switzerland; 2 Faculty of Physics, University of Vienna, Vienna, Austria

**Keywords:** Nanophotonics and plasmonics, Optical manipulation and tweezers, Statistical physics

## Abstract

Rare transitions between long-lived metastable states underlie a great variety of physical, chemical and biological processes. Our quantitative understanding of reactive mechanisms has been driven forward by the insights of transition state theory and in particular by Kramers’ dynamical framework. Its predictions, however, do not apply to systems that feature non-conservative forces or correlated noise histories. An important class of such systems are active particles, prominent in both biology and nanotechnology. Here, we study the active escape dynamics of a silica nanoparticle trapped in a bistable potential. We introduce activity by applying an engineered stochastic force that emulates self-propulsion. Our experiments, supported by a theoretical analysis, reveal the existence of an optimal correlation time that maximises the transition rate. We discuss the origins of this active turnover, reminiscent of the much celebrated Kramers turnover. Our work establishes a versatile experimental platform to study single particle dynamics in non-equilibrium settings.

## Abstract (web summary)

The famous Kramers turnover describes the extent of friction at which the transition rate of a small particle trapped in a bistable potential becomes a maximum. Militaru et al. present a version of this phenomenon pertaining to active colloids driven by non-conservative forces.

## Introduction

Transitions between long-lived states are important for the understanding of chemical reactions^1,2^, transitions between bistable configurations^3,4^, protein folding^5,6^, motion of ligands in proteins^7^, diffusion in solids through different domains^8^, nuclear fission^9^ and current switching in Josephson junctions^10^. The transition rate, also called reaction rate, represents a central measure in this context, quantifying the frequency of transitions unfolding in metastable and multistable systems. The first steps towards a quantitative understanding of reaction rates date back to the 19th century^1,2,11,12^, yet it was only in 1940 that Kramers developed the dynamic framework^13^ widely used to this day. He considered a Brownian particle moving in a bistable potential and derived limiting expressions for high and low friction. Kramers realized that the transition rate constant disappears in both friction limits, and thus inferred the existence of a global maximum at some intermediate value of the damping, an aspect known today as the Kramers turnover^14–17^. It was only recently that the Kramers turnover has been measured in a single experimental system^18^.

Kramers’ framework and its extensions^19–22^, however, are a result of equilibrium dynamics and thus no longer apply in the presence of non-conservative forces. A particularly interesting example in which such forces are important is active matter. In active matter, the constituents draw on internally stored or externally supplied energy to propel themselves and drive the system out of equilibrium. Self-propulsion gives rise to various intriguing collective phenomena, such as swarming and orientation phase transitions^23^. Even on an individual particle basis, self-propulsion holds great potential for applications in microscopic transport and sensing. The perhaps simplest model for active matter is the active particle—a particle subjected to thermal noise, dissipation, and to a self-propelling force of constant magnitude and Brownian orientation^24–26^. These self-propelling agents arise in various contexts such as Janus particles^25,27^, micro- and nanorobots^28,29^, motion of bacteria^30,31^, and active transport of biological macromolecules^27,32,33^. Understanding and controlling active particles thus represents a challenge of great importance in nanotechnology and medical sciences^34^.

Previous attempts towards investigating the transition rates of active matter, crucial for their transport through constrictions and interfaces, are constrained to overdamped dynamics or to an activity induced by a velocity-dependent damping^35–39^. Yet any type of movement in low-density media, for instance dilute gases, is heavily affected by inertial effects^40^. The Kramers turnover in particular represents an interesting example of inertial effects on transition phenomena. Furthermore, advancements in nanotechnology require the examination of automated and stochastic self-propulsion in various environmental conditions. The transition rate of active particles in the underdamped regime is thus a key question which has remained surprisingly unexplored to date.

In this work, we experimentally investigate and theoretically analyse the transition rate of an active particle in a bistable potential over a wide range of frictions. We implement an active particle by applying an engineered stochastic force to an optically levitated nanoparticle. Our setup allows us to span both the overdamped and the underdamped motional regimes. We observe an additional turnover as a function of the decorrelation time of the propulsion’s orientation. This activity-related turnover is of a different nature from its passive counterpart, and the two are shown to coexist in a two-dimensional parameter space. The experimental observations are in quantitative agreement with theoretical results and numerical simulations.

## Results

### Experimental system

**Fig. 1:**
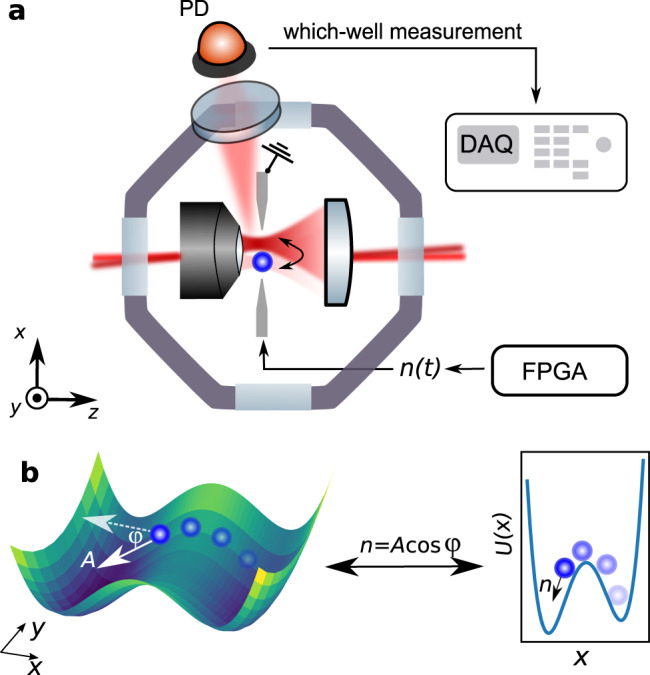
Model under study and experimental setup. **a** Experimental setup. We create a bistable optical potential for a charged silica nanoparticle by focusing two cross-polarized beams through a 0.80 NA objective. We implement a which-well measurement by collecting the laterally scattered light with a photodiode (PD), whose signal is recorded with a Data Acquisition Card (DAQ). Owing to the different polarizations in the two wells, the intensity measured by the photodiode is a binary signal that indicates in which potential well the particle resides. The particle carries a finite net electric charge and is made active along the *x*-direction with an electrostatic force generated by a voltage applied across lateral electrodes. This voltage is generated by a field-programmable gate array (FPGA). **b** A two-dimensional active particle in a potential landscape that is bistable along the *x*-direction and harmonic along *y*. The active particle is propelled by a force of constant magnitude *A* and Brownian orientation *φ*. The dimensions *x* and *y* are decoupled and the *x*-motion is equivalent to a particle moving in a one-dimensional bistable potential under the influence of a time-dependent force $n(t)=A\cos \varphi (t)$.

The experimental setup is shown in Fig. 1a. A charged silica nanoparticle of nominal diameter 136 nm is trapped in a bistable optical potential. The bistable potential is realized by focusing two cross-polarized and frequency-shifted Gaussian beams through a high NA objective (wavelength *λ* = 1064 nm). The two foci lie along the *x*-axis and their distance is controlled by a careful alignment of the relative angle between the beams. To implement a direct which-well measurement, we introduce a photodetector to monitor the light scattered by the particle in a direction perpendicular to the optical axis. Owing to the mutually orthogonal polarization of the two beams, together with the radiation pattern of a linear dipole (which emits no radiation along its axis), the recorded signal displays jumps as the particle transitions from one well to the other. The traces recorded by the photodetector are used for the study of the transition rates presented throughout this work.

We apply an external electrostatic force that mimics the behaviour of active propulsion parallel to the potential’s bistability direction. The voltage signal used to apply the active force is generated by a FPGA (see the “Methods” section). Throughout this work, we study a particle actively propelled by a force of constant magnitude *A*, called activity, with stochastically changing direction *φ*. After projecting the active force onto the bistability direction *x*, the motion of the particle is described by the following Langevin equations:

1a $$m\ddot{x}+m{{{\Gamma }}}_{0}\dot{x}+{\partial }_{x}U(x)=A\cos \varphi +{{\mathcal{F}}}_{\text{th}},$$

1b $$\dot{\varphi }=\sqrt{2{D}_{{\rm{R}}}}{\eta }_{{\rm{R}}}(t).$$

The position *x* evolves in time under the influence of a frictional force proportional to the damping coefficient Γ_0_, a conservative trapping force arising from the bistable potential *U*(*x*), and thermal noise at temperature *T* related to the friction via the fluctuation-dissipation theorem ${{\mathcal{F}}}_{\text{th}}=\sqrt{2m{{{\Gamma }}}_{0}{k}_{{\rm{B}}}T}{\eta }_{\text{th}}(t)$^41^. The two mutually uncorrelated white noises *η*_th_ and *η*_R_ individually satisfy the properties $\left\langle \eta (t)\right\rangle =0$ and $\left\langle \eta (t)\eta (t^{\prime} )\right\rangle =\delta (t-t^{\prime} )$. The damping coefficient Γ_0_ can be tuned by changing the pressure of the vacuum chamber^42^. The orientation of the active force follows overdamped and purely diffusive dynamics associated with the rotational diffusivity *D*_R_. In the following, we use $n=A\cos \varphi$ to refer to the one-dimensional active force. The system is illustrated schematically in Fig. 1b. Equations (1a) and (1b) are an accurate description of many artificially synthesized swimmers, above all Janus particles^23,25,27^. While realistic biological swimmers tend to follow more complex dynamics of the propulsion direction^23,33^, it is instructive to use this simple model to gain insight into the basic phenomenology of active escape dynamics.

**Fig. 2:**
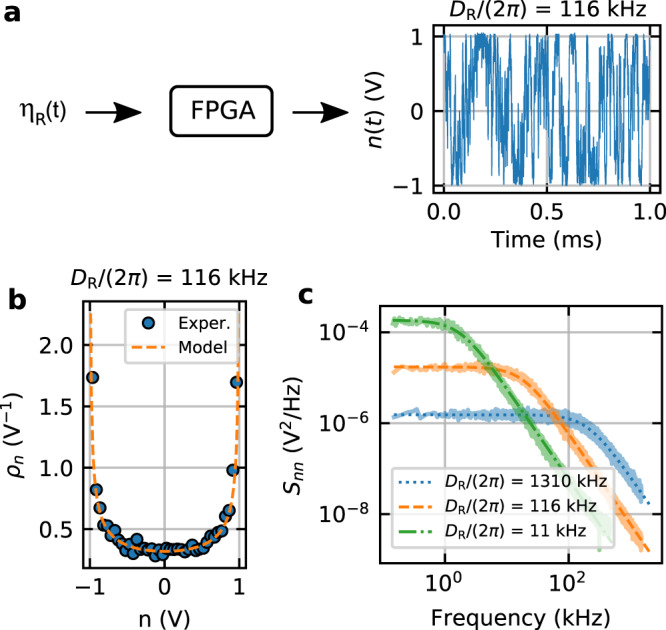
Statistical properties of the active force. The values shown are electric signals which act on the particle through the Coulomb force. **a** Transformation of white Gaussian noise through an FPGA into the active force $n=A\cos \varphi$ of Eqs. (1a) and (1b) (*D*_R_ = 2*π* × 116 kHz). **b** Probability density of the realized active force in **a**. The arcsine distribution (dashed line) is expected from the projection onto one dimension of a constant force with fluctuating direction. **c** Example spectra of active forces for three different values of *D*_R_. Panels **b** and **c** highlight the non-Markovian and non-Gaussian nature of active propulsion. We refer to Supplementary Note 6 for a derivation of the theoretical curves shown as dashed lines.

Figure 2 shows the measured characteristics of the active force, i.e., of the voltage output by our custom-programmed FPGA (see Supplementary Note 6). Specifically, in Fig. 2a we show an example time trace of the active force for *D*_R_ = 2*π* × 116 kHz. Figure 2b depicts the histogram of the trace in Fig. 2a, and Fig. 2c shows three examples of the power spectral density (PSD) *S*_*nn*_ for different rotational diffusivities. In stark contrast to the thermal fluctuations induced by the surrounding gas, the activity’s noise history is non-Gaussian and coloured.

### Transition rate measurement

**Fig. 3:**
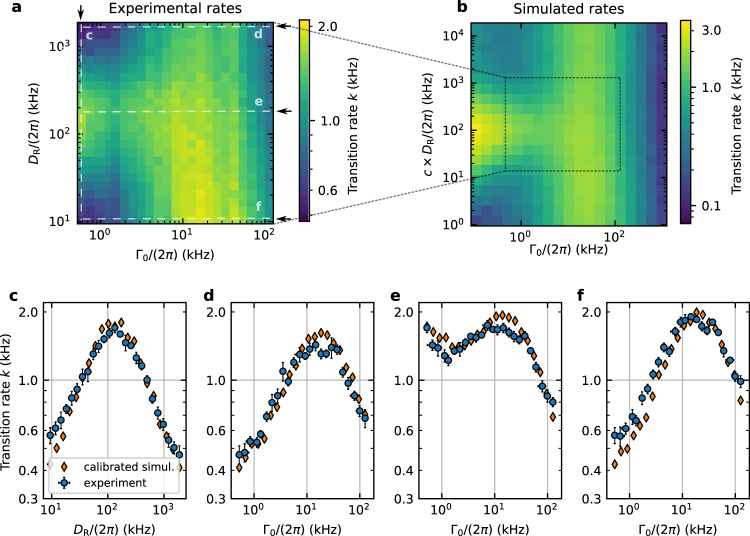
Experimental and numerical transition rates. **a** Experimental transition rate constant *k* as a function of rotational diffusivity *D*_R_ and translational damping Γ_0_. The dashed lines parallel to the arrows refer to the four cuts shown in **c**–**f**. At low Γ_0_ we observe the active turnover along the *D*_R_-axis. For fixed *D*_R_ we recover a Kramers-like turnover. **b** Computationally obtained transition rate constant (activity *A* = 9.35 fN). The simulated *D*_R_-axis is rescaled by the calibration factor *c* = 2.24, as described in the main text. The transition rate constant agrees within roughly 10% with the calibrated numerical estimates. **c** Active turnover. Vertical cut at Γ_0_ = 2*π* × 523 Hz of the experimental (blue circles) and of the simulated (orange diamonds) transition landscapes. **d** Horizontal cut at *D*_R_ = 2*π* × 1.8 MHz. High rotational diffusivity in conjunction with sufficiently high friction leads to a recovery of inactive dynamics and of the Kramers turnover. **e** Horizontal cut at *D*_R_ = 2*π* × 166 kHz, roughly the position of the active turnover. Starting at high Γ_0_ and moving towards lower values, we initially encounter the Kramers-like turnover mainly arising from thermal transitions. Passing the local minimum, a further steady increase in the transition rate is induced by weaker damping facilitating the active propulsion. **f** Horizontal cut at *D*_R_ = 2*π* × 9 kHz. As *D*_R_ approaches zero the active force becomes a modification to the potential with an effectively constant *φ*. The resulting Kramers-like turnover results from an average over the corresponding rate constants. The rate extraction and error bars (standard errors of the mean) are described in the “Methods” section.

The central quantity of interest in the present study is the transition rate constant *k*, i.e., the typical frequency of transitions between the metastable states of the potential. It is extracted from the decaying autocorrelation of the which-well measurement (see the “Methods” section). Figure 3a showcases the transition rate constant as a function of rotational diffusivity *D*_R_ and translational damping Γ_0_. Each data point stems from a 30 s long trajectory with fixed pressure and rotational diffusivity. We observe two perpendicular lines of cross-section maxima. The vertical line of maxima appears at roughly Γ_0_ = 2*π* × 20 kHz and corresponds to the Kramers turnover^18^. The second, horizontal one emerges at *D*_R_ = 2*π* × 166 kHz and represents the central result of this work: an activity-induced turnover. We additionally depict four cross-sections highlighting the active turnover at Γ_0_ = 2*π* × 523 Hz (Fig. 3c), its passive Kramers counterpart at *D*_R_ = 2*π* × 1.8 MHz (Fig. 3d), a cut along the rotational diffusivity *D*_R_ = 2*π* × 166 kHz that corresponds to the active turnover (Fig. 3e), and the Kramers-like turnover at *D*_R_ = 2*π* × 9 kHz (Fig. 3f). The prominence of the active turnover decreases with increasing damping Γ_0_, blends into the Kramers turnover and vanishes for very high dampings.

In order to shed light on the nature of the active turnover, we implemented a numerical reconstruction of the observed transition rate landscape. The numerical reconstruction, displayed in Fig. 3b, aims at recovering the landscape’s key features using three fit parameters: barrier height, activity, and distance between potential wells. The numerical reconstruction is in quantitative agreement with the experimental data throughout the observed parameter space. In addition to the three fit parameters, we introduce a calibration factor *c* for the rotational diffusivity, needed to bridge the one-dimensional simulation and the more complicated three-dimensional reality. Generally, in higher-dimensional systems it is possible for the particle to prefer different transition channels when driven by the activity or by thermal fluctuations. Such a multiplicity cannot occur in the one-dimensional model of Eqs. (1a) and (1b), it can however easily appear in the experiment, caused for instance by a slight misalignment between potential minima and activity axes. Additional details about the numerical reconstruction can be found in the “Methods” section and Supplementary Note 5.

### Activity-driven transition events

The structure of the observed rate landscape in Fig. 3 is surprisingly rich. Nonetheless, its individual features can be understood on the basis of a few intuitive arguments stemming from a more rigorous analysis presented in the Supplementary Notes 3 and 4. The existence of a maximum along the Γ_0_-axis is analogous to the Kramers turnover in passive systems. Its renewed emergence supports the continued validity of Kramer’s predictions even in non-conservative setups, extending their range of applicability. Next, we focus on the aspects arising specifically due to the presence of active propulsion. With the angle evolving according to Brownian motion, see Eqs. (1a) and (1b), active propulsion can be interpreted as a stochastic force characterized by an exponentially decaying autocorrelation $\left\langle \cos \varphi (t)\cos \varphi (t^{\prime} )\right\rangle =\exp (-{D}_{{\rm{R}}}| t-t^{\prime} | )/2$. This relation identifies the rotational diffusivity as a measure of the characteristic timescale during which the active force’s orientation does not change appreciably: the persistence time ${\tau }_{{\mathrm{{A}}}}={D}_{{\rm{R}}}^{-1}$. It represents the key to reveal the mechanisms underlying the active turnover. To this end, let us conduct a simple thought experiment.

We start from the case of low rotational diffusivity: if the particle’s orientation persists for a very long time, it is possible to consider the barrier to be modified by an additional tilt to the potential of $-Ax\cos \varphi$ (with constant *φ*). This modification to the barrier height facilitates the transition in one direction while hampering the reverse one. As *A* increases, this trend becomes more apparent and at some point we would almost necessarily have to wait for the active force to change its sign to observe the next transition. For very large *A* and small *D*_R_ we expect to observe a single transition at best and remain stuck in the temporarily (or practically eternally) favoured well. The rate constant needs to effectively vanish in this extreme scenario.

Very high values of *D*_R_ affect the system in a fundamentally different manner: if the activity’s direction becomes completely decorrelated on the timescale of small positional displacements, its presence only increases the translational diffusivity in magnitude. In other words, fast rotating active forces raise the particle’s effective temperature. Over the course of a barrier transition the orientation *φ* assumes all its possible realizations and can no longer persistently push the particle over the barrier. The transition rate constant is nevertheless enhanced given that larger diffusivities allow to scale barriers more easily in general. Specifically, as *D*_R_ is decreased from infinity this effect becomes gradually stronger since the random displacements caused by the activity increase in size on average. This implies a higher effective temperature at lower *D*_R_ and subsequently higher transition rate constants *k*. The extreme scenario of infinitely fast rotation, on the other hand, will result in no net change of the effective translational diffusion and a recovery of inactive dynamics.

Finally, it is reasonable to expect a maximum in the transition rate constant if the persistence time is similar to the characteristic duration necessary to transverse the barrier, namely the average transition path time. Then one typically retains the active force’s aid during the whole transition, without inhibiting the reverse reaction any longer than necessary. Incidentally, this line of thinking closely follows Kramers’ original argument for the existence of the passive turnover as a consequence of two opposing monotonic trends. Here, the position of the maximum is closely tied to the average transition path time as well, roughly emerging when the energy dissipated during the transition is comparable to the thermal energy *k*_B_*T*. The particle’s trajectory then experiences thermal decorrelation that is sufficiently fast to avoid subsequent recrossings (which do not contribute to the rate) without losing the benefit of a more persistent direction of the velocity.

With the timescales of energy dissipation and orientation decorrelation determined by separate parameters, the location of the passive and active turnover on their respective axis become largely independent of changes in the remaining variable, i.e., *D*_R_ or Γ_0_, respectively. This results in the cross-section maxima of Fig. 3 forming two approximately perpendicular lines. The last prominent feature, the turnover’s gradual disappearance at large Γ_0_, constitutes the property easiest to explain. Any sufficiently high friction can serve to slow down and practically arrest the particle’s movement, leading to the rate constant’s general decrease as one steps farther into the overdamped regime. Conversely, raising *A* allows the active noise to compete against strong dissipative forces, making the phenomenon relevant for overdamped dynamics as well.

## Discussion

Our experimental platform can be adapted in the future to address various theoretical concepts arising in stochastic thermodynamics involving non-equilibrium systems and correlated noise histories. For instance, with the inclusion of a linear position measurement across a wide region of space, we can extend the model to a full three-dimensional activity rather than a one-dimensional projection. This setup would enable a rigorous investigation on the statistics of transition path times and the emergence of different pathways preferred by active and thermal transitions as a function of the activity^43^. With the inclusion of a position feedback we can generate more complex kinds of position-dependent and time-dependent activities, which have recently been shown to have intriguing tactic properties^44^. Furthermore, our externally applied stochastic force is not inherently constrained to mimic active propulsion, but rather allows for the introduction of any desired noise history into the system. This versatility opens up experimental opportunities in the field not tied to activity, such as fluctuation theorems in the presence of coloured noise.

In summary, we have extended the analysis of the kinetics of transitions in a bistable system to active particles covering both the regimes of overdamped and underdamped motion. We have observed a turnover in the transition rate that arises as the persistence time of the active particle gradually increases from values much shorter to values much longer than the dynamical timescales of the system. A simplified one-dimensional description is sufficient to replicate the rich phenomenology of our experimental findings, and is capable of generating quantitatively consistent predictions. A full closed-form description that bridges the low with the high rotational diffusivity limits remains an open question tied to the complexity of the underlying Fokker–Planck equation. Furthermore, it would be interesting to study general systems with exponentially decaying or other types of memory. Explicitly investigating the impact of various characteristic memory timescales on transition rates may be helpful to uncover the mechanisms behind rare events in non-Markovian systems, possibly even extending the generality of the phenomenology observed in this work.

## Methods

### Experimental setup

The frequency shift (80 MHz) between the two beams is introduced by an acousto-optic modulator (AOM) acting on a *λ* = 1064 nm wavelength laser. The AOM also controls the power of the two beams, equal to 70 ± 1 (110 ± 1) mW for the *x*- (*y*-) polarized beam. After the focus, the beams are recollimated and separated with a polarizing beam splitter (PBS). We use a standard homodyne position measurement on the *x*-polarized beam to characterize the potential curvatures^45^. The curvatures are estimated through time–frequency analysis: the estimated values are *ω*_1_ = 2*π* × (73.0 ± 0.5) kHz for the *x*-polarized well and *ω*_2_ = 2*π* × (82 ± 4) kHz for the *y*-polarized well. The proportionality coefficient between damping and pressure is inferred via the PSD of the particle when the *y*-polarized laser is turned off^46^. Specifically, the PSD of a harmonic oscillator is given by a Lorentzian whose width equals the damping coefficient.

### Active force

The active force is realized with a field-programmable gate array (FPGA) that takes as input a white noise created by a function generator. The FPGA integrates Eq. (1b) to generate the active force as output. We exploit the net charge carried by the particle and apply the active force electrostatically through two electrodes mounted along the bistability direction^47^. The experimental value of the activity can be determined from the response of the particle to a known modulated voltage. Within 50% accuracy due to the mass uncertainty, we estimated *A* = 6.8 fN. A potential barrier height Δ*U* of a few *k*_B_*T* and diffraction limited width Δ*x* ≈ *λ*/2 gives rise to conservative forces with a typical magnitude of Δ*U*/Δ*x* ≈ 8 fN. In order to induce a measurable effect the activity needs to be of the same order of magnitude, as is the case here.

### Transition rate estimation

Rate coefficients appear within the framework of the eponymous rate equations. This type of differential equation aims to describe the evolution of local concentrations or population fractions in a set of states {*A*, *B*, … } subjected to reactive transitions. Our case, a bistable system with population fractions *c*_*A*_ and *c*_*B*_, represents a particularly simple example described by ${\dot{c}}_{A}=-{k}_{AB}{c}_{A}+{k}_{BA}{c}_{B}=k({c}_{A,{\rm{eq}}}-{c}_{A})$. With the asymmetry between rate constants *k*_*AB*_ and *k*_*BA*_ attributable to the stationary concentration *c*_*A*,eq_ = *k*_*BA*_/(*k*_*AB*_ + *k*_*BA*_), the transition rate *k* represents the dynamical parameter governing the speed of equilibration. Within the approximation of rate equations, *c*_*A*_(*t*) = (*c*_*A*_(0) − *c*_*A*,eq_)e^−*kt*^ + *c*_*A*,eq_ approaches its equilibrium value exponentially and the rate can be extracted from sufficiently long sample trajectories^18^. The rates in Fig. 3, more specifically, are extracted from 30 s long trajectories. We split each trajectory into 10 segments and compute the average rate and its standard deviation.

### Numerical reconstruction

The simulation results complementing our experimental findings stem from the optimization of an effective, one-dimensional potential along with the activity A. We use a bistable, piece-wise parabolic potential, continuous up to its first derivative and tune its barrier width/curvature *ω*_B_ and height *h*. Equations (1a) and (1b) are then numerically integrated for values of Γ_0_ and *D*_R_ spaced on a logarithmic grid. We employ the OVRVO integrator devised by Sivak et al. for the particle’s propagation^48^. The curvatures of the well-parabolas respect the experimentally determined particle frequencies. The obtained transition landscapes are compared to the experimental reference w.r.t. a small number of effective quantities related to the active and passive turnovers, respectively: maximum height, its position on the respective axis, and turnover width at half maximum, all evaluated on a logarithmic scale. We proceed to locate the parameter set of *h*, *ω*_B_, and *A* that leads to the minimal deviation in the aforementioned measures on a discrete, linearly spaced grid of desired resolution. Starting from cautious a-priori estimates of these values, we rely on a bisection-like approach to locate said minimum. To simplify this procedure, note that at high *D*_R_ one essentially recovers inactive dynamics, allowing us to optimize *h* and *ω*_B_ independently of *A*. Additional details on the simulation and optimization procedure can be found in Supplementary Note 5.

## Supplementary information

Supplementary Information

## Data Availability

The relevant datasets generated and analysed throughout this work are available from the corresponding author upon reasonable request. Source data for all figures shown in the main text and Supplementary Material are provided with the paper.

## Source data

Source Data

## References

[1] Arrhenius S (1889). Über die Reaktionsgeschwindigkeit bei der Inversion von Rohrzucker durch Säuren. Z. Phys. Chem..

[2] van’t Hoff JH (1884). Etudes de dynamique chimique. Recl. Trav. Chim. Pays-Bas.

[3] Ricci F (2017). Optically levitated nanoparticle as a model system for stochastic bistable dynamics. Nat. Commun..

[4] Landauer R, Swanson JA (1961). Frequency factors in the thermally activated process. Phys. Rev..

[5] Šali A, Shakhnovich E, Karplus M (1994). How does a protein fold?. Nature.

[6] Frauenfelder H, Sligar SG, Wolynes PG (1991). The energy landscapes and motions of proteins. Science.

[7] Beece D (1980). Solvent viscosity and protein dynamics. Biochemistry.

[8] D’Agliano EG, Kumar P, Schaich W, Suhl H (1975). Brownian motion model of the interactions between chemical species and metallic electrons: bootstrap derivation and parameter evaluation. Phys. Rev. B.

[9] Bohr N, Wheeler JA (1939). The mechanism of nuclear fission. Phys. Rev..

[10] Silvestrini P, Pagano S, Cristiano R, Liengme O, Gray KE (1988). Effect of dissipation on thermal activation in an underdamped Josephson junction: first evidence of a transition between different damping regimes. Phys. Rev. Lett..

[11] Eyring H (1935). The activated complex in chemical reactions. J. Chem. Phys..

[12] Hänggi P, Talkner P, Borkovec M (1990). Reaction-rate theory: fifty years after Kramers. Rev. Mod. Phys..

[13] Kramers HA (1940). Brownian motion in a field of force and the diffusion model of chemical reactions. Physica.

[14] Grabert H, Linkwitz S (1988). Effect of time-delayed friction on the escape from a metastable well. Phys. Rev. A.

[15] McCann LI, Dykman M, Golding B (1999). Thermally activated transitions in a bistable three-dimensional optical trap. Nature.

[16] Turlot E (1989). Escape oscillations of a Josephson junction switching out of the zero-voltage state. Phys. Rev. Lett..

[17] Troe J (1986). Elementary reactions in compressed gases and liquids: from collisional energy transfer to diffusion control. J. Phys. Chem..

[18] Rondin L (2017). Direct measurement of Kramers turnover with a levitated nanoparticle. Nat. Nanotechnol..

[19] Hanggi P, Mojtabai F (1982). Thermally activated escape rate in presence of long-time memory. Phys. Rev. A.

[20] Pollak E (1986). Theory of activated rate processes: a new derivation of Kramers’ expression. J. Chem. Phys..

[21] Pollak E, Grabert H, Hänggi P (1989). Theory of activated rate processes for arbitrary frequency dependent friction: solution of the turnover problem. J. Chem. Phys..

[22] Pollak E, Ankerhold J (2013). Improvements to Kramers turnover theory. J. Chem. Phys..

[23] Bechinger C (2016). Active particles in complex and crowded environments. Rev. Mod. Phys..

[24] Volpe G, Gigan S, Volpe G (2014). Simulation of the active Brownian motion of a microswimmer. Am. J. Phys..

[25] Howse JR (2007). Self-motile colloidal particles: from directed propulsion to random walk. Phys. Rev. Lett..

[26] Romanczuk P, Bär M, Ebeling W, Lindner B, Schimansky-Geier L (2012). Active Brownian particles: from individual to collective stochastic dynamics. Eur. Phys. J. Spec. Top..

[27] Ghosh PK, Misko VR, Marchesoni F, Nori F (2013). Self-propelled janus particles in a ratchet: numerical simulations. Phys. Rev. Lett..

[28] Golestanian R, Liverpool TB, Ajdari A (2007). Designing phoretic micro- and nano-swimmers. New J. Phys..

[29] Sundararajan S, Lammert PE, Zudans AW, Crespi VH, Sen A (2008). Catalytic motors for transport of colloidal cargo. Nano Lett..

[30] Berg, H. C. *E. coli in Motion* (Springer, 2014).

[31] Gejji R, Lushnikov PM, Alber M (2012). Macroscopic model of self-propelled bacteria swarming with regular reversals. Phys. Rev. E.

[32] Brenner H (1990). Macrotransport processes. Langmuir.

[33] Bressloff PC, Newby JM (2013). Stochastic models of intracellular transport. Rev. Mod. Phys..

[34] Ebbens SJ, Howse JR (2010). In pursuit of propulsion at the nanoscale. Soft Matter.

[35] Geiseler A, Hänggi P, Schmid G (2016). Kramers escape of a self-propelled particle. Eur. Phys. J. B.

[36] Caprini L, Marini Bettolo Marconi U, Puglisi A, Vulpiani A (2019). Active escape dynamics: the effect of persistence on barrier crossing. J. Chem. Phys..

[37] Schaar K, Zöttl A, Stark H (2015). Detention times of microswimmers close to surfaces: influence of hydrodynamic interactions and noise. Phys. Rev. Lett..

[38] Pohlmann L, Tributsch H (1997). Self-organized electron transfer. Electrochim. Acta.

[39] Burada PS, Lindner B (2012). Escape rate of an active Brownian particle over a potential barrier. Phys. Rev. E.

[40] Scholz C, Jahanshahi S, Ldov A, Löwen H (2018). Inertial delay of self-propelled particles. Nat. Commun..

[41] Kubo R (1966). The fluctuation-dissipation theorem. Rep. Prog. Phys..

[42] Beresnev SA, Chernyak VG, Fomyagin GA (1990). Motion of a spherical particle in a rarefied gas. Part 2. Drag and thermal polarization. J. Fluid Mech..

[43] Zijlstra N, Nettels D, Satija R, Makarov DE, Schuler B (2020). Transition path dynamics of a dielectric particle in a bistable optical trap. Phys. Rev. Lett..

[44] Geiseler A, Hänggi P, Marchesoni F (2017). Self-polarizing microswimmers in active density waves. Sci. Rep..

[45] Gieseler J, Deutsch B, Quidant R, Novotny L (2012). Subkelvin parametric feedback cooling of a laser-trapped nanoparticle. Phys. Rev. Lett..

[46] Hebestreit E (2018). Calibration and energy measurement of optically levitated nanoparticle sensors. Rev. Sci. Instrum..

[47] Frimmer M (2017). Controlling the net charge on a nanoparticle optically levitated in vacuum. Phys. Rev. A.

[48] Sivak DA, Chodera JD, Crooks GE (2014). Time step rescaling recovers continuous-time dynamical properties for discrete-time Langevin integration of nonequilibrium systems. J. Phys. Chem. B.

