# Supplementary material for: Escape dynamics of active particles in multistable potentials
Source: Nat Commun. 2021 Apr 27;12:2446. doi: 10.1038/s41467-021-22647-6 (PMC8079420; doi:10.1038/s41467-021-22647-6)
Supplement: Supplementary file 1 — Supplementary Information [file 41467_2021_22647_MOESM1_ESM.pdf]

Supplementary Information:
Escape dynamics of active particles in multistable potentials

A. Militaru,[1,†] M. Innerbichler,[2,†] M. Frimmer,[1]
F. Tebbenjohanns,[1] L. Novotny,[1] and C. Dellago[2,*]

[1]*Photonics Laboratory, ETH Zurich, CH-8093 Zurich, Switzerland*

[2]*Faculty of Physics, University of Vienna, 1090 Vienna, Austria*

SUPPLEMENTARY NOTE 1: EXTRACTION OF RATE COEFFICIENTS

Let us explicitly consider a two-state system characterized by the concentrations/population fractions c_A and c_B. In the context of single particle systems one can define $c_A(t)$ via an ensemble of a large number of replicas. Then, $c_A(t)$ corresponds to the fraction of replicas in state A at time t. Subjected to reactive dynamics, c_A evolves according to the rate equation

$$\dot{c}_A = -k_{AB}c_A + k_{BA}c_B = k_{BA} - (k_{AB} + k_{BA})c_A. \tag{1}$$

The rate constants k_{AB} and k_{BA} respectively prescribe the speed of the reaction from A to B and from B to A. Population/particle conservation enforces a constraint of the form $\sum_X c_X = 1$, used above to arrive at the rightmost expression. It is straightforward to determine the solution as

$$c_A(t) = c_A(0)e^{-(k_{AB}+k_{BA})t} + \frac{k_{BA}}{k_{AB}+k_{BA}}\left[1 - e^{-(k_{AB}+k_{BA})t}\right]. \tag{2}$$

Hence, the populations approach their equilibrium values $c_{A,\mathrm{eq}}$ and $c_{B,\mathrm{eq}}$ exponentially with a decay-time controlled by the sum of the rate constants $k = k_{AB} + k_{BA}$. We underline this point by reformulating the above equation in terms of the equilibrium/stationary state concentration $c_{A,\mathrm{eq}}$

$$c_A(t) = \left[c_A(0) - c_{A,\mathrm{eq}}\right] e^{-kt} + c_{A,\mathrm{eq}}, \tag{3}$$

where the equilibrium population is given by $c_{A,\mathrm{eq}} = k_{BA}/(k_{AB} + k_{BA})$. The rate constants can therefore be extracted from the exponential decay/growth of the concentration. For the initial condition $c_A(0) = 1$, the population $c_A(t)$ can be interpreted as the probability of finding the particle in well A at time t provided it was located there at time $t = 0$. Contact between the phenomenological rate equation, i.e. Supplementary Equation (1), and the experimental time traces of the system can be made by considering the indicator function h_A, which is a function equal to unity inside well A and zero otherwise. Then, the average $\langle h_A(t)\rangle$ of the indicator function corresponds to the population $c_A(t)$.

---

† These two authors contributed equally.

* e-mail: christoph.dellago@univie.ac.at

In terms of the indicator function h_A, the conditional probability to find the system in A at time t, provided it was there at time $t_0 = 0$, can be expressed using the time correlation function of the population as $\langle h_A(0)h_A(t)\rangle / \langle h_A\rangle$. Here, the average is taken over a long (equilibrium/stationary state) trajectory. Assuming that for sufficiently long times the kinetics of the system is described by the rate equation, one obtains

$$\langle h_A(0)h_A(t)\rangle = c_{A,\mathrm{eq}}(1 - c_{A,\mathrm{eq}})e^{-kt} + c_{A,\mathrm{eq}}^2, \tag{4}$$

$$\langle \Delta h_A(0)\Delta h_A(t)\rangle = \left\langle \Delta h_A(0)^2\right\rangle e^{-kt}, \tag{5}$$

where Δh_A designates $h_A - \langle h_A\rangle$ and we took advantage of the relations $\langle h_A\rangle = c_{A,\mathrm{eq}}$ and $h_A^2 = h_A$. Consequently, one can extract the rate coefficient k by performing a single parameter fit to the (normalized) autocorrelation function. In reality, however, the rate equation only represents a convenient simplification of the system at hand and deviations from purely exponential decays are expected, for instance due to short-time correlations. In order to avoid including short-time correlations in the rate estimations, we fit Supplementary Equation (5) only over time scales where their effect has disappeared.

SUPPLEMENTARY NOTE 2: ACTIVITY AND EFFECTIVE BARRIER HEIGHT

To supplement the experimental findings delineated in the main part of the document, we provide additional context by studying the transition dynamics theoretically and computationally. For clarity, we direct our focus mainly on the phenomenology and select a particularly simple and frequently used form of the potential, i.e.

$$U(x) = h\left(1 - \frac{x^2}{\sigma^2}\right)^2. \tag{6}$$

The parameter h gives the height of the barrier and the minima of the symmetric potential are located at $\pm\sigma$. All quantities with dimensions of inverse time are subsequently given in terms of $k_0 = t_0^{-1} = \sqrt{k_\mathrm{B}T/m\sigma^2}$. We investigate the shape of the resulting transition rate landscape as function of the translational damping Γ_0 and rotational diffusivity D_R. We generate and analyse individual long trajectories for each set of Γ_0 and D_R on a logarithmically spaced grid, whilst all other parameters remain fixed. Further details about the simulation are delineated in Supplementary Note 5.

Some illustrative examples of the resulting rate landscapes with various activity values are depicted in Supplementary Fig. 1. Starting our discussion from low (almost vanishing) activity, one observes the conventional Kramers turnover only. As one would expect in this case, the transition rate rises to its maximum at some moderate value of Γ_0 and remains virtually unaffected by the active contributions. Stronger activities, however, lead to data exhibiting some interesting features: a second line of maxima appears in the direction of the rotational diffusion axis D_R. This line seems to stop abruptly at the position of the Kramers turnover. Further increasing A results in an evermore prominent active turnover, completely superseding its original counterpart at its horizontal position. The Kramers turnover has not disappeared, yet its magnitude is far too small to be seen on a linear scale. The active turnover is unable to push past the limit imposed by the passive one and disappears gradually as the damping is increased. However, for even higher A, it becomes possible for the active turnover to stretch farther into the overdamped regime, simultaneously becoming more prominent at lower Γ_0 values.

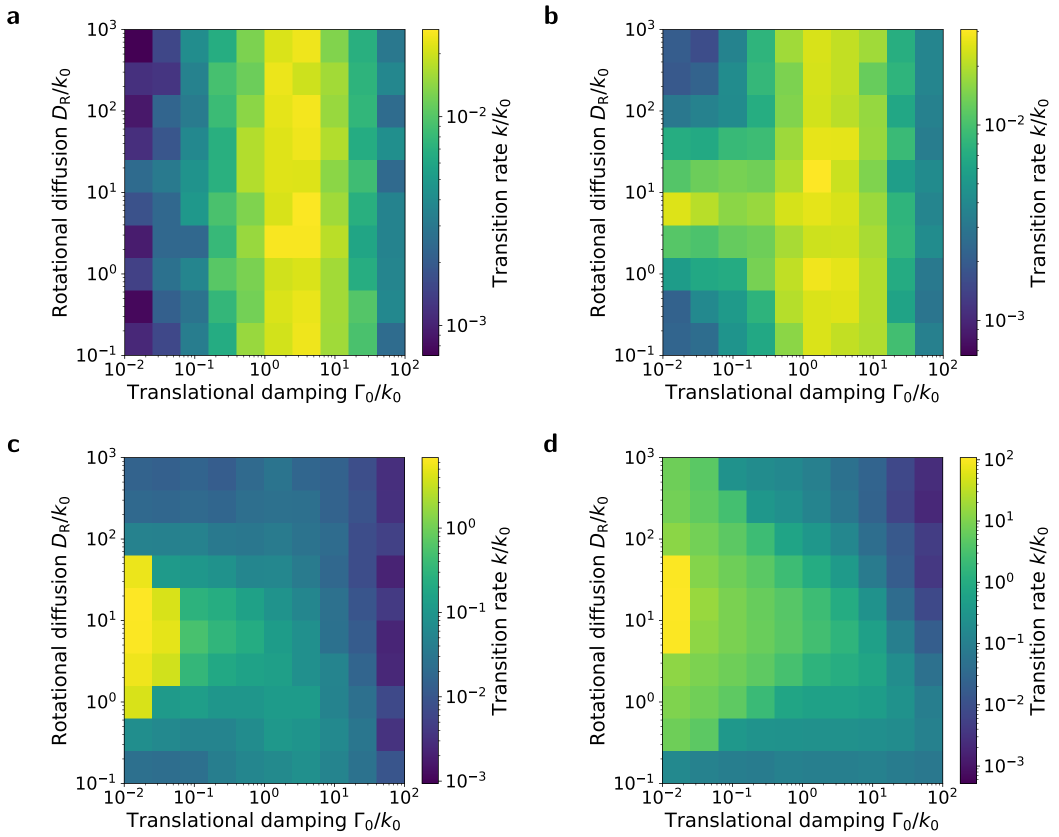

Supplementary Figure 1. **Transition rate as a function of the translational damping and rotational diffusivity for four activity magnitudes.** From **a** to **d**, the used activity values read $A\sigma/k_BT = 0.25$, 1.00, 4.00, 16.0. As A increases the active turnover appears and becomes increasingly more prominent, whereas the Kramers turnover retains its magnitude. Simulation parameters: barrier height $h = 4k_BT$, time step $10^{-2}t_0$, trajectory length per data point $T = 2 \times 10^4 t_0$.

We have provided an intuitive motivation of the features arising due to active propulsion in the main text. In the following, we proceed to investigate the active turnover more quantitatively. Following Kramers' example, we demonstrate the existence of a turnover by examining the limits of high and low rotational diffusivity individually. Instead of directly jumping to these limiting scenarios, however, it is instructive to discuss the activity's non-equilibrium nature.

Because it continuously feeds energy into the system, active propulsion constitutes an example of a non-conservative force. As such, the system's stationary state $p_s(x, v, \varphi)$ is not described by the Boltzmann distribution. The stationary state becomes explicitly dependent on the damping and rotational diffusivity, distinctly setting the underlying physics apart from the properties generating the Kramers turnover: in conservative settings, the rate exhibits a maximum at moderate friction due to purely dynamical effects, while the stationary probability density in phase space is independent of the damping Γ_0.

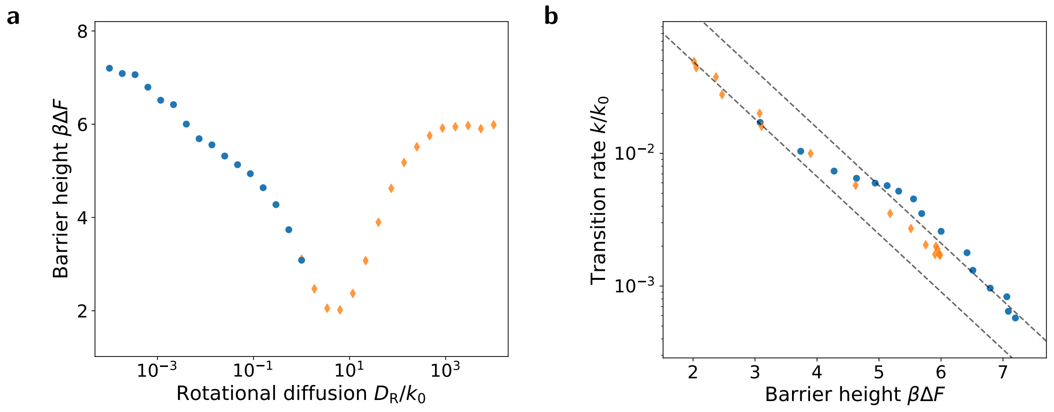

Supplementary Figure 2. **Barrier height and impact on transition rates.** **a**, Free energy barrier height $\beta\Delta F$ as a function of rotational diffusivity D_{R}. **b**, Transition rate k as a function of the free energy barrier height $\beta\Delta F$. All data points stem from trajectory simulations in the quartic potential of Supplementary Equation (6) with potential barrier height $h = 6k_{\mathrm{B}}T$, activity $A = 3k_{\mathrm{B}}T/\sigma$, and at a damping of $\Gamma_0 = 0.2k_0$. The trajectory length per point equals $T = 10^6 t_0$. The showcased data encompasses two separate sets of simulations for the high and low D_{R} regime only differing in the size of time step Δt. We used $\Delta t = 10^{-2}t_0$ for the low D_{R} part (blue circles) and $\Delta t = 10^{-3}t_0$ for the fast rotation section (orange diamonds), as a finer discretization is needed to thoroughly resolve the evolution of the angle. The two gray lines accompanying the generalized Arrhenius plot indicate the slope expected theoretically. A switch in the rate's prefactor seems to emerge around $4k_{\mathrm{B}}T$ - about a factor two in magnitude - not attributable to stationary effects. Nonetheless, the data's general consistency with the theoretical approximation points to the barrier height's central role in determining the transition rate k.

While usually introduced in equilibrium thermodynamics and statistical physics, it is useful to define the free energy F for non-conservative settings analogously as the negative natural logarithm of the stationary state via $\beta F = -\ln p_{\mathrm{s}}(x, v, \varphi)$ [1]. Adopting the view of transition state theory [2], the rate depends exponentially on the height of the free energy barrier ΔF, i.e. it is proportional to the probability to reside atop the barrier

$$k \propto e^{-\beta\Delta F}. \tag{7}$$

Even small modifications of ΔF affect the rate landscape considerably. Studying the variation of ΔF as a function of A, Γ_0, and D_{R} leads to a more quantitative understanding of the emergence of the active turnover. The Arrhenius plot depicted in Supplementary Fig. 2 serves to establish $\beta\Delta F$ as the key quantity in shaping the activity-dominated part of the rate landscape. The fact that only comparatively small deviations from the postulated exponential decay arise suggests that most relevant effects are indeed summarized into this single measure. This circumstance constitutes the basis of the fairly simple quantification approaches laid out below.

Our physical model is described by a set of Langevin equations. Therefore the evolution of its phase space density $p(x, v, \varphi, t)$ is governed by the associated Fokker-Planck equation (FPE), with the velocity diffusion coefficient given by $D = \Gamma_0 k_{\mathrm{B}}T/m$

$$\partial_t p(x, v, \varphi) = -v\partial_x p + \partial_v \left(\Gamma_0 v + \frac{U'(x)}{m} - \frac{A}{m}\cos\varphi\right) p + D\partial^2_{vv} p + D_{\mathrm{R}}\partial^2_{\varphi\varphi} p. \tag{8}$$

The stationary distribution p_s and free energy F are in principle obtained by imposing $\partial_t p = 0$. Unfortunately, as of now a closed-form solution of the resulting FPE remains elusive, forcing us to resort to numerical or approximate treatments. The upcoming derivations for the high and low D_R limits are inherently based on the FPE, although some aspects are understood more easily in the Langevin picture. The theories successfully reproduce the transition rates obtained computationally, capturing the essential aspects to affect the free energy barrier height.

SUPPLEMENTARY NOTE 3: HIGH ROTATIONAL DIFFUSIVITY LIMIT

Let us first consider the limit of large D_R, in which the persistence time of the active force is far shorter than all remaining physical timescales. This effectively implies that before the particle changes its position in phase space by an appreciable amount, the direction of its active propulsion completely decorrelates, allowing it to perform several stochastic rotations on the spot. Therefore, at the timescale of translational motion the active noise behaves as if subsequent realizations were statistically independent, effectively putting it into the same memory class as white noise. Based on this reasoning, a modification of the diffusion constant or, equivalently, the introduction of an effective temperature provides an adequate approximation of the impact of the active force on the system. To justify this approximation quantitatively, let us quickly consider the limit $D_\mathrm{R} \to \infty$. For an orientation changing infinitely fast, the angle distribution must be uniform at each point in reduced phase space (x, v). The effective dynamics in reduced phase space then results from replacing all φ-dependent terms with their local averages. The expectation value of the active force $A\cos\varphi$ vanishes given a uniformly distributed angle, leading us to the same equation of motion as in the absence of activity:

$$m\dot{v} = -m\Gamma_0 v - U'(x) + \sqrt{2m\Gamma_0 k_\mathrm{B} T}\eta(t). \tag{9}$$

Next, we consider the equation of motion at the translational timescale for a finite (but still very high) rotational diffusivity. We emphasise that for fast rotational diffusion it is possible to choose a duration Δt that allows for substantial changes in φ whilst keeping x and v effectively constant. The deterministic displacement in x and v throughout the time step Δt should then be virtually unaffected by the details of the noise history in-between. This allows us to investigate the displacements attributed to the (active) noise terms without the presence of any deterministic drifts due to potential forces and friction. Our argument should even work in the presence of multiplicative noise as the current point in position- and velocity-space, for all intents and purposes, turns into a parameter.

The total velocity displacement due to stochastic forces $\Delta_\mathrm{s} v$ over Δt can be expressed as

$$\Delta_\mathrm{s} v = \int_0^{\Delta t} dt \left(\frac{A}{m} \cos\varphi(t) + \sqrt{2D}\eta(t) \right). \tag{10}$$

We consider velocity displacements because the active turnover resides primarily in the underdamped regime. The present argumentation can be applied to the overdamped approximation as well. We will do so when studying its disappearance for increasing Γ_0. We compute the mean

square displacement associated with Supplementary Equation (10) to obtain

$$\begin{aligned}\left\langle \Delta_\mathrm{s} v^2 \right\rangle &= \left\langle \int_0^{\Delta t} dt \int_0^{\Delta t} dt' \left(\frac{A}{m} \cos\varphi(t) + \sqrt{2D}\eta(t) \right) \left(\frac{A}{m} \cos\varphi(t') + \sqrt{2D}\eta(t') \right) \right\rangle = \\ &= \int_0^{\Delta t} dt \int_0^{\Delta t} dt' \frac{A^2}{m^2} \left\langle \cos\varphi(t)\cos\varphi(t') \right\rangle + 2D \left\langle \eta(t)\eta(t') \right\rangle = \\ &= \int_0^{\Delta t} dt \int_0^{\Delta t} dt' \left(\frac{A^2}{2m^2} e^{-D_\mathrm{R}|t-t'|} + 2D\delta(t-t') \right). \end{aligned} \tag{11}$$

The expectation values of the mixed terms disappear due to statistical independence, splitting them into a product of expectation values equal to zero. The autocorrelation of the white noise yields a Dirac-delta by definition and the cosine term is derived in Supplementary Note 6. The final result reads

$$\begin{aligned}\left\langle \Delta_\mathrm{s} v^2 \right\rangle &= 2D\Delta t + \frac{A^2}{m^2 D_\mathrm{R}} \left(\Delta t + \frac{1}{D_\mathrm{R}} \left(e^{-D_\mathrm{R}\Delta t} - 1 \right) \right) = \\ &= 2\left(D + \frac{A^2}{2m^2 D_\mathrm{R}} \right) \Delta t + \mathcal{O}(D_\mathrm{R}^{-2}). \end{aligned} \tag{12}$$

Comparing Supplementary Equation (12) with the inactive dynamics, we obtain an effective translational diffusion coefficient equal to $D_\mathrm{eff} = D + A^2/(2D_\mathrm{R})$, hereby neglecting the small terms of order D_R^{-2}. These higher order terms emerge from the fact that the active noise is inherently not a Gaussian white noise even in the limit considered. For instance, white noise can induce arbitrarily large displacements throughout Δt, whereas active noise has an upper bound in magnitude at $A\Delta t$. Nonetheless, these differences become negligible for very high D_R. In terms of an effective temperature one obtains

$$D_\mathrm{eff} = \frac{\Gamma_0 k_\mathrm{B} T_\mathrm{eff}}{m} = \frac{\Gamma_0 k_\mathrm{B} T}{m} \left(1 + \frac{A^2}{2m\Gamma_0 k_\mathrm{B} T D_\mathrm{R}} \right). \tag{13}$$

Attributing all relevant modifications between the active and inactive stationary distribution to this effective temperature leads us to the relation

$$\beta \Delta F = \frac{\Delta U}{k_\mathrm{B} T_\mathrm{eff}}, \tag{14}$$

where ΔU denotes the energy barrier of the trapping potential.

At this point we are able to approximate the high rotational diffusivity part of the landscape with a single parameter fit of the form

$$k(D_\mathrm{R}) = c_0 e^{-\Delta U / k_\mathrm{B} T_\mathrm{eff}(D_\mathrm{R})}. \tag{15}$$

The applicability of our findings is highlighted in Supplementary Fig. 3. The average kinetic energy K shown is related to the effective temperature via $K = k_\mathrm{B} T_\mathrm{eff}/2$ and is approximated very well by Supplementary Equation (13). It remains quite consistent even in close proximity to the active turnover, where the assumptions of our simplification no longer apply. A similar quality is achieved in the parameter-fit showcased in Supplementary Fig. 3b. The D_R-dependence beyond the active turnover seems well-explained by an Arrhenius-like estimate along with the effective temperature provided.

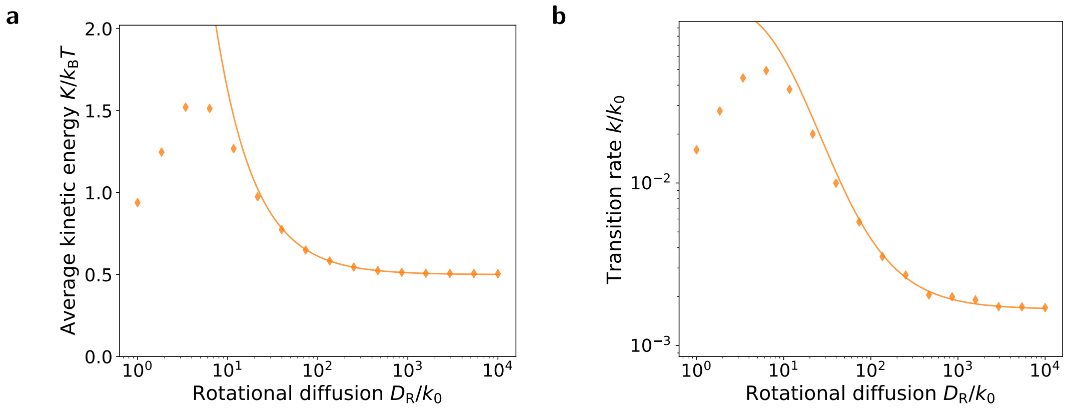

Supplementary Figure 3. **Effective temperature model in the fast rotation regime.** Average kinetic energy (**a**) and transition rate constant (**b**) as a function of the rotational diffusion. The parameters used are the same as those of Supplementary Fig. 2. The solid lines represent the theoretical approximations of Supplementary Equation (13), with the approximation for the kinetic energy being parameter-free. The solid curve on the right is fitted to the range of high D_R only, amounting to a vertical displacement on the logarithmic scale. Both theoretical predictions are consistent with the data within their designated regime. Fit parameter: $c_0 = 0.648k_0$.

Termination in the Overdamped Regime

The termination of the active turnover at higher dampings Γ_0 shown in Supplementary Fig. 1 can be derived in a similar vein to what we discussed in the previous section. The central premise leading to the activity's inclusion in the diffusive term consists of the persistence time being far smaller than the translational timescale. Whether this goal is achieved by speeding up the rotation or inhibiting translational movement is irrelevant. Increasing the friction serves to gradually arrest the dynamics and we end up with the same result as before even without considering the Langevin equation's overdamped limit explicitly. In what follows, the diffusivity still pertains to the velocity and not to the position ($D = \Gamma_0 k_B T/m$). The expression for the effective temperature

$$k_B T_{\text{eff}} = k_B T \left(1 + \frac{A^2}{2m\Gamma_0 k_B T D_R}\right) \tag{16}$$

gives a quantitative estimate about the influence of the active forces on the overall rate landscape at high Γ_0, gradually decaying as A^2/Γ_0 approaches zero. Irrespective of the activity A, one can always select a sufficiently high damping for the active turnover to effectively disappear. On the other hand, raising A necessitates a quadratic increase of Γ_0 to suppress its effects to the same extent in this approximation. This aspect supports the further expansion of the active turnover into the overdamped regime with increasing A as well as the rate's overall increase, clearly visible in Supplementary Fig. 1d.

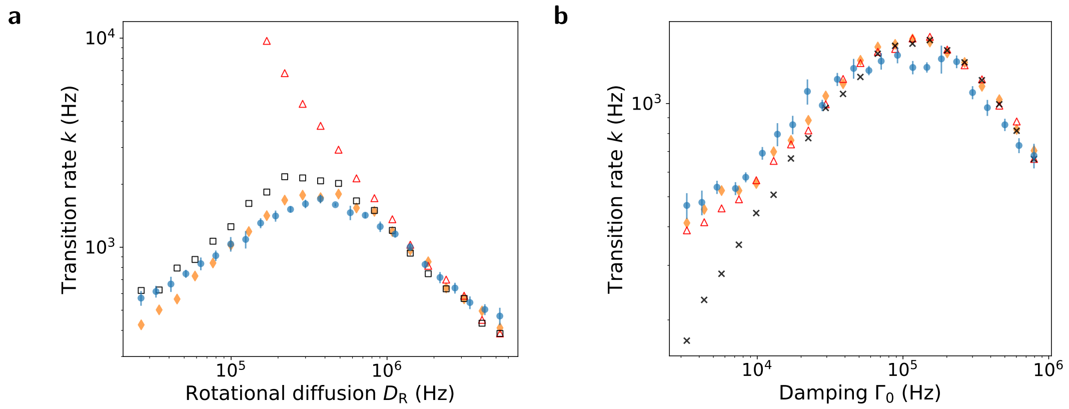

Supplementary Figure 4. **Comparison of experimental and computationally reconstructed transition rates with the effective temperature description.** **a**, Active turnover at low damping ($\Gamma_0 = 2\pi \times 523\,\mathrm{Hz}$). **b**, Kramers turnover at high rotational diffusivity (experimental value $D_\mathrm{R} = 2\pi \times 1.8\,\mathrm{MHz}$). The panels correspond to Figs. 3c and 3d of the main text. Blue circles: Experimental rates with standard deviation of the mean as error bars. The D_R-axis is shifted by the calibration factor c, see Supplementary Note 5. Orange diamonds: Computational replication of the experimental rate landscape as described in Supplementary Note 5. Red triangles: Inactive particle simulations with ambient temperature given by Supplementary Equation (16). Black squares: Inactive particle simulation with effective temperature extracted from the average kinetic energy obtained from our simulations (orange diamonds). Black crosses: Inactive particle simulation at constant ambient temperature.

Effective Temperature and Inactive Particle Simulations

Before moving on to our treatment of the slow rotation regime, we provide some additional evidence for the applicability of the effective temperature model along with an example for the suppression of activity-induced effects at higher damping Γ_0. Specifically, we consider a part of the experimental data and its computational replication, which is fully described in Supplementary Note 5. The following analysis relies on simulations of an inactive particle in the reconstructed potential at various temperatures, and the results should therefore also be compared with the reconstructed rate landscape to check for remaining discrepancies. The main motivation for our reliance on explicit simulations rather than predictions based on the Arrhenius-ansatz of Supplementary Equation (15) comes from the experimental potential's rather low energy barrier of around $4k_\mathrm{B}T$. At this point finite barrier effects become evermore important in determining the rate. A numeric treatment incorporates these corrections naturally and can be conducted without any free parameters.

Supplementary Fig. 4 shows the results of our inactive particle simulations along with two of the highlighted rate landscape cross-sections appearing in Fig. 3 of the main text, corresponding to the active and the Kramers turnover (panels c and d), respectively. Let us first discuss the rates of an inactive particle at an ambient temperature as predicted by Supplementary Equation (16). We find excellent agreement between our simulations with explicit activity and those obtained on basis of the effective temperature model in the high D_R regime. This consistent behaviour should not come as a surprise - fundamentally the effective temperature serves as an accurate treatment of the fast varying rotational degree of freedom. Quantities derived from the slowly

changing variables of the system, i.e. position and velocity, should be replicated truthfully even by this simplified inactive representation - this includes the transition rate. Nevertheless, as D_R decreases, the model's central premise - rotation being faster than all other dynamical timescales - starts to break down, leading to the clear discrepancies first appearing in the active turnover's vicinity. We shall discuss these shortly.

The damping-dependent set of rates (Supplementary Fig. 4b), on the other hand, remains consistent with the reference data throughout the whole range of Γ_0 values investigated. Due to the already high D_R, the effective temperature is sufficient to explain the activity-induced modifications to the rate. Here, not slow rotation but weak damping leads to the activity's prominent heating effect. To facilitate comparison we have included a rate curve of a particle immersed in a bath of constant ambient temperature. The active heating evidently slows the rate's decrease when diminishing Γ_0, anticipating the steady growth that would follow afterwards. Conversely, on the high-Γ_0 side even before reaching the proximity of the Kramers-predicted maximum, T_eff closely approaches the environmental temperature T, causing all simulation results to merge. In this regime activity plays no part in determining the slow variables' dynamics. In other words, activity-induced effects become negligible provided sufficiently high friction and constant D_R.

Finally, let us discuss the departure from the reference data seen in Supplementary Fig. 4a, artificially caused by the boundless growth of Supplementary Equation (16) with decreasing D_R. As mentioned, this approximation is no longer applicable once other dynamical timescales become faster than the rotational diffusion. Nonetheless, even at this point one can sensibly ask whether the rate's decrease towards the left can be viewed as a consequence of the decline in average kinetic energy, or whether other effects need to be taken into account for a proper quantitative understanding of its behaviour. Extracting the average kinetic energy as a function of D_R from the reference data, we conduct simulations of a passive particle at the corresponding temperature. We find that this approach extends the applicability of the effective temperature picture only within the immediate proximity of the active turnover. Although sufficient to predict the general trend as a function of D_R including a maximum, the extracted rates still differ appreciably from their reference values at low D_R. This implies that other mechanisms contribute significantly in determining the rate of slowly rotating particles, aspects we will address in the next section.

SUPPLEMENTARY NOTE 4: LOW ROTATIONAL DIFFUSIVITY LIMIT

Infinitely Slow Rotation

In the extreme case in which the rotational diffusivity is infinitesimal, we can consider the angle φ as a parameter and the active force as a modification of the potential equal to $-Ax\cos\varphi$. This effective potential exhibits different left and right barrier heights that are functions of φ - we denote them with $\Delta U_\mathrm{L}(\varphi)$ and $\Delta U_\mathrm{R}(\varphi)$ for the left and right well respectively. Analogously, we define the left and right transition rate as $k_\mathrm{L}(\varphi)$ and $k_\mathrm{R}(\varphi)$.

First of all, let us introduce an expression for the effective transition rate derived from an ensemble containing all possible modified potentials: for a fixed angle the probability to stay in the left well decays per definition like $e^{-k_\mathrm{L}(\varphi)t}$. The long-time decay of a cumulative measure or population that has components individually decaying exponentially is dominated by the slowest

rate present. Nonetheless, we need to extract an effective rate that circumvents addressing these individual components directly - a point of view that summarizes all potential tilts into a single rate equation.

To this end, consider the average transition probability $p_{\mathrm{L,cm}}$ at low waiting times Δt (i.e. the probability to observe a jump between wells within this duration)

$$p_{\mathrm{L,cm}} = \int_0^{2\pi} d\varphi \, \left(1 - e^{-k_{\mathrm{L}}(\varphi)\Delta t}\right) p_{\mathrm{L}}(\varphi) =: 1 - e^{-k_{\mathrm{L,eff}}\,\Delta t}, \tag{17}$$

where p_L denotes the probability density to find the particle in orientation φ under the constraint that it currently resides in the left well. The integral introduces an effective rate k_{eff} that generally depends on Δt. Nonetheless, at Δt much smaller than the multiplicative inverse of all rates present, one can conveniently expand the above expression to obtain

$$k_{\mathrm{L,eff}} = \int_0^{2\pi} d\varphi \, k_{\mathrm{L}}(\varphi) p_{\mathrm{L}}(\varphi), \tag{18}$$

equalling the standard expectation value. The short time behaviour (i.e., shorter than any rate and of course diffusion timescales) of the left and right well concentrations is then consistently represented by the original ODE as

$$\dot{c}_{\mathrm{L}} = -k_{\mathrm{L,eff}}\, c_{\mathrm{L}} + k_{\mathrm{R,eff}}\, c_{\mathrm{R}}. \tag{19}$$

In the case of infinitely slow rotational diffusion we are in the fortunate position to know a closed form of the marginal angular distribution. Under this condition each angle can be treated as a separate case, allowing us to recycle the result shown in Supplementary Equation (2). Up to a normalization factor it reads

$$p_L(\varphi) \propto \frac{k_{\mathrm{R}}(\varphi)}{k_{\mathrm{L}}(\varphi) + k_{\mathrm{R}}(\varphi)}. \tag{20}$$

In its current form, our result is unfortunately independent of D_{R}. In the next section we introduce a straightforward variant of the above model that amends this shortcoming and discuss the conditions for its applicability.

Reaction-Diffusion Model

Rate equations are typically not concerned with details about the velocity and position beyond classifying the current state, allowing us to discard spurious details. We can thus integrate the irrelevant variables over the phase space portion corresponding to the state. Proceeding with this line of thought transforms the FPE into a reaction-diffusion equation as shown in the following.

Without loss of generality, we place the boundary between the two wells at $x = 0$ and define the left-well population as

$$c_{\mathrm{L}}(\varphi, t) := \int_{-\infty}^{\infty} dv \, \int_{-\infty}^{0} dx \, p(x, v, \varphi, t). \tag{21}$$

We apply the same integrations to the FPE (Supplementary Equation (8)) to obtain:

$$\partial_t c_{\mathrm{L}} = D_{\mathrm{R}}\partial^2_{\varphi\varphi}c_{\mathrm{L}} + \int_{-\infty}^{\infty} dv \int_{-\infty}^{0} dx \left[-v\partial_x p + \partial_v\left(\Gamma_0 v + \frac{U'(x)}{m} - \frac{A}{m}\cos\varphi\right)p + D\partial^2_{vv}p\right] =$$
$$= D_{\mathrm{R}}\partial^2_{\varphi\varphi}c_{\mathrm{L}} - \int_{-\infty}^{\infty} dv\; vp(x=0,v,\varphi,t). \quad (22)$$

Under the reasonable assumption that the probability distribution decays faster than any linear (typically polynomial) function, all terms containing a derivative of the velocity drop out. A similar idea was applied to the lower limit of the position integral. Alternatively, one can assume a confined/compact initial condition, which generates a time-dependent distribution that vanishes at infinity anyhow. Our expressions are still exact at this point under these conditions. We shall now proceed to apply physically motivated approximations for the remaining integral term of Supplementary Equation (22), which describes the net probability flux through the dividing surface.

We exclude stochastic recrossings where the particle velocity changes its sign in the immediate proximity of the dividing surface. These cover a very small fraction of all reactive paths and contribute practically nothing to the integral for one of two reasons: either the velocity multiplied to the local distribution is already very small or the sign reversal becomes extremely unlikely. From this standpoint, almost all instances of (appreciably large) negative velocities on the barrier come from the right well and vice versa, splitting the integral into two terms associated with the particle origin and thus proportional to the respective local concentration c_{L} or c_{R}.

In the next step, we use the fact that since the rotational diffusivity is small, one can treat the angle as constant at least over the duration of the average transition path time. The probability to visit the barrier top is then approximately proportional to the Boltzmann factor of the effective potential including its linear tilt due to active propulsion $U_{\mathrm{eff}}(x,\varphi) = U(x) - Ax\cos\varphi$. In the context of this conservative-like dynamics, the velocity part of the distribution should factorize as usual and appear in terms of the kinetic energy. The velocity integral thus becomes an additional prefactor, similar to the normalization of the local Boltzmann distribution. Therefore we arrive at

$$\partial_t c_{\mathrm{L}}(\varphi,t) = D_{\mathrm{R}}\partial^2_{\varphi\varphi}c_{\mathrm{L}} - z_L e^{-\beta\Delta U_{\mathrm{eff},L}}c_{\mathrm{L}} + z_R e^{-\beta\Delta U_{\mathrm{eff},R}}c_{\mathrm{R}}$$
$$= D_{\mathrm{R}}\partial^2_{\varphi\varphi}c_{\mathrm{L}} - k_{\mathrm{L}}(\varphi)c_{\mathrm{L}} + k_{\mathrm{R}}(\varphi)c_{\mathrm{R}}. \quad (23)$$

The velocity parts and normalizations are absorbed into the dynamic prefactors z_L, z_R. In a symmetric potential these quantities become equivalent if we neglect their weak φ-dependence, and once again constitute the only parameter(s) of the model. Together with the Boltzmann factor we can identify their product as angle-dependent rates, resulting in a reaction-diffusion equation much simpler than the original FPE as it depends on only two variables instead of the original four.

Deriving the analogous expression for the right well and using the constraint of an overall uniform marginal angle distribution in the interval $[0, 2\pi)$, we end up with a single equation governing the stationary state

$$D_{\mathrm{R}}\partial^2_{\varphi\varphi}c_{\mathrm{L}} = [k_{\mathrm{L}}(\varphi) + k_{\mathrm{R}}(\varphi)]c_{\mathrm{L}} - \frac{1}{2\pi}k_{\mathrm{R}}(\varphi). \quad (24)$$

In presence of the boundary conditions of positivity and periodicity, this ordinary linear differential equation has, in principle, a unique solution. It can even be further simplified to a system

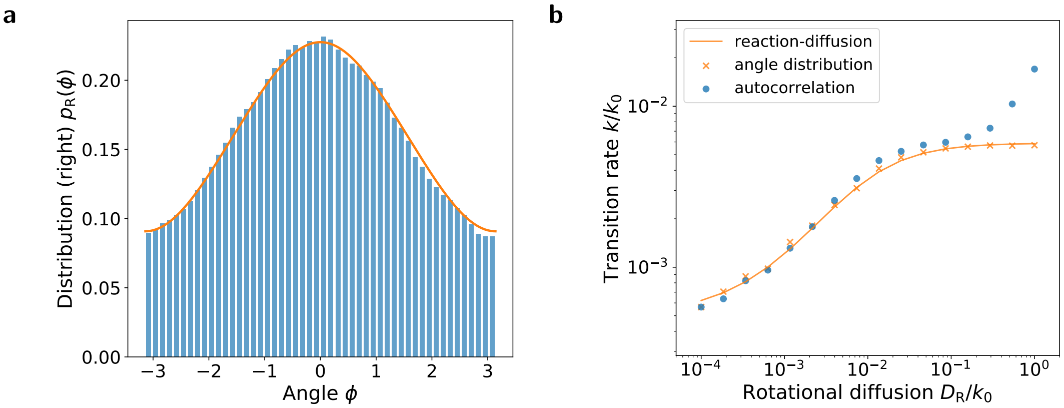

Supplementary Figure 5. **Reaction-diffusion model in the slow rotation regime.** **a**, Example of a φ-distribution under the condition that the particle sojourns in the right well ($D_{\mathrm{R}} = 1.35 \times 10^{-2} k_0$). **b**, Comparison of transition rates extracted with the autocorrelation method (blue circles) against Boltzmann-factor averages using the observed distributions (orange crosses). The same simulation parameters were used as in the high D_{R} investigation, but the time step was increased to obtain longer trajectories at the same expense (each by a factor ten). Additional estimates computed via the reaction-diffusion model are included as well, depicted as solid orange lines in the distributions and rate estimates. Using a constant prefactor $z_L = z_R = 0.3k_0$ as the only parameter in the model, we attempt to reproduce the real angle distributions and therefrom estimated rates. The assumption of constant prefactors z_L, z_R appears to hold within the regime of validity of the averaging approximation.

of first-order ODEs by defining $w_{\mathrm{L}} := \partial_\varphi c_{\mathrm{L}}$

$$\frac{d}{d\varphi}\begin{pmatrix} c_{\mathrm{L}} \\ w_{\mathrm{L}} \end{pmatrix} = \begin{pmatrix} 0 & 1 \\ k_{\mathrm{L}} + k_{\mathrm{R}} & 0 \end{pmatrix}\begin{pmatrix} c_{\mathrm{L}} \\ w_{\mathrm{L}} \end{pmatrix} - \frac{1}{2\pi}\begin{pmatrix} 0 \\ k_{\mathrm{R}} \end{pmatrix}. \tag{25}$$

For finite (and low) rotational diffusivity, the normalized solution of Supplementary Equation (25) represents the stationary distribution to calculate the effective rate as seen in Supplementary Equation (18). To reiterate, the central requirement for the validity of our effective model is only a matter of the rotational diffusivity's relative smallness and our expressions finally include D_{R} explicitly. Unfortunately, even this simplified model does not seem to have an easily accessible closed-form solution due to the complex form of our rate function. The theory's performance can be checked by means of computational data, however, highlighted in Supplementary Fig. 5. The showcased example of an angle distribution is fully in line with our expectations, with angles favouring transitions into the respective well becoming increasingly more prominent at lower D_{R}.

Furthermore, Supplementary Fig. 5b depicts the slow rotation part of the rate landscape from last section. Besides increasing the time step and trajectory length by a factor ten, the simulation parameters have been left as they were. The angular distributions along with the active modifications of the potential are used to compute the rate up to a common prefactor (amounts to a simple displacement in logarithmic scale, the value of the lowest point is made to coincide with the autocorrelation data). Our predictions turn out to be very accurate for an extensive regime of rotational diffusivity values and capture the rate's initial increase up until the theory

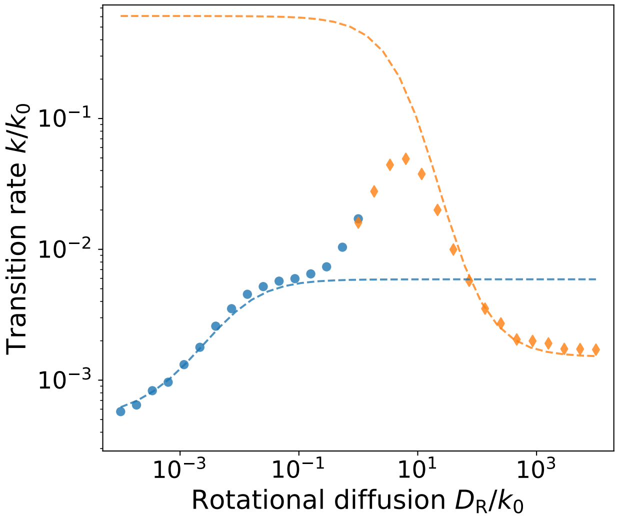

Supplementary Figure 6. **Transition rates at low damping.** Side-by-side depiction of the high and low D_R data and plots (see Supplementary Figs. 3b, 5b). Outside the regime of the turnover itself, our models make very accurate predictions about the landscape's shape, capturing its key features.

reaches its high D_R plateau. At that point we are faced with a situation where our initial premises and conditions fall apart, making this discrepancy fully expected. The theory seems like an adequate approximation up until one to two orders of magnitude before reaching the active turnover, falling short of the fast rotation limit in terms of accurate coverage.

Finally, Supplementary Fig. 6 showcases the results of both models alongside another, accurately replicating the landscape over all possible D_R safe for the immediate proximity of the active turnover. Both approaches only rely on the dynamic prefactor as parameter, corresponding to simple displacements of the curves on a logarithmic scale. The good consistency between theory and data hints at both theories capturing the essential details affecting the rate.

SUPPLEMENTARY NOTE 5: NOTES ON THE SIMULATION

In this section, we summarize the general outline of the simulations conducted. We propagate trajectories in one spatial dimension by using the OVRVO integrator first devised by Sivak et al. [3] and based on the well-known Liouville framework for symplectic integrators. Compared to a standard velocity Verlet integrator, this method additionally encompasses appropriate Ornstein-Uhlenbeck steps placed at the beginning and the end of each iteration to act as the system's thermostat. Each data point in the transition landscapes results from a trajectory of duration T generated with time step Δt, specified in the description of the respective figures. As all rate coefficients shown were extracted from long trajectories and reflect the stationary state, the initial condition becomes irrelevant. For completeness, however, let us note we generally start in one of the minima with a velocity drawn from the Maxwell-Boltzmann distribution.

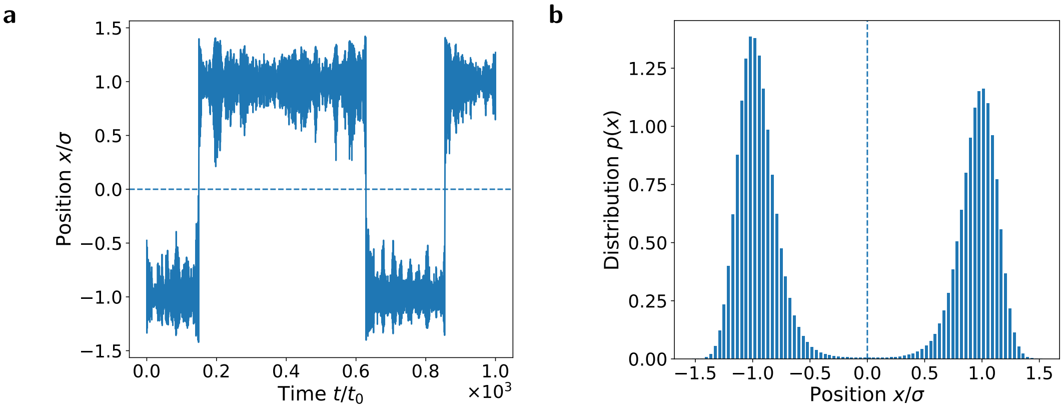

Supplementary Figure 7. **Sample trajectory of a reactive system.** **a**, Segment of a trajectory in a bistable trap. **b** Full-length trajectory histogram, belonging to the data shown in Supplementary Fig. 3 at $D_{\mathrm{R}} = 4.64 \times 10^2 k_0$. The dashed line demonstrates the dividing surface's location.

Next, we proceed to delineate the analysis methods applied to both numerical and experimental trajectories.

The first step of any rate extraction algorithm consists in the selection of an appropriate dividing surface. Let us direct our attention to Supplementary Fig. 7, showcasing a segment of a simulated trajectory. The peaks in the distribution corresponding to the metastable states are separated by a range of low probability. For signals of this kind, the extracted rate does not depend sensibly on the dividing surface's exact location. Shifting it slightly to the left or right influences our results only negligibly, which is why a straightforward selection method fully serves our purposes. We locate the approximate position of the maxima left and right by generating histograms with a prescribed bin width and place our surface in the minimum between those points. This approach relies on sufficiently smooth histograms to yield appropriate results, which all investigated trajectories (experimental or computational ones) were able to provide.

Having placed the dividing surface, our signal is converted into an indicator function to subsequently evaluate its normalized autocorrelation, an example of which is shown in Supplementary Fig. 8. We invoke the Wiener-Khinchin theorem and compute all autocorrelations via FFTs for computational efficiency. The initial behaviour of our autocorrelations is very well-represented by a simple exponential decay except a brief period at the very beginning attributed to short-time correlations. The curves' roughness increases as the autocorrelation approaches zero as a consequence of fewer uncorrelated indicator pairs, but can be diminished by analyzing longer trajectories (i.e., gathering more statistics). Excluding the initial non-exponential deviation, we fit an exponential of the form $c_0 e^{-kt}$ to these curves, limiting the regime to the part of the normalized autocorrelation larger than a prescribed threshold, here 0.10. The parameter k should then correspond to the sum of left and right transition rates, completing the extraction.

Uncertainties have only been computed for the experimental data presented in the main text, evaluated by splitting the trajectories into ten segments each. We extract the rate for each segment and compute the standard deviation of the mean as our error estimates.

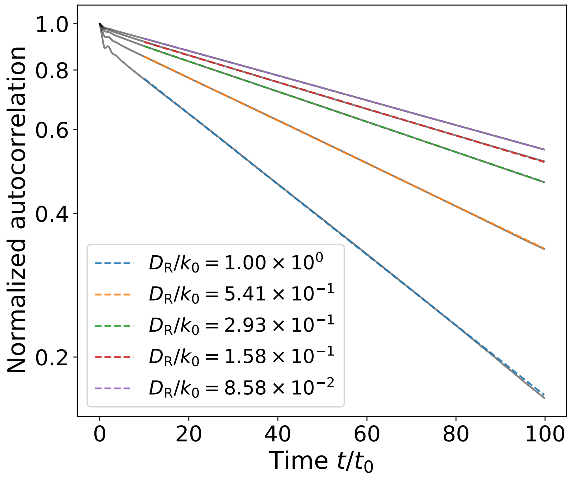

Supplementary Figure 8. **Normalized autocorrelation functions for five values of the rotational diffusion.** The fits (dashed lines) exhibit an excellent agreement with the computational data (gray lines). At very short timescales deviations from the predicted exponential decay occur, which are excluded from the fitting regime.

Replication Study

In this section we describe how we computationally replicated the experimental findings seen in the main text, specifically the rate landscape visualized in Fig. 3. Such an endeavour provides additional insight by unearthing differences and physical contributions not captured by the simple Langevin model. Since the signal mainly serves to distinguish between wells rather than providing an accurate position at any point of time, it is not inherently well-suited to infer the experimental potential. Unable to directly extract the shape of the physical potential from the signals, we opt for a model that allows to prescribe all well-known information along with the remaining parameters to be optimized.

The key quantities of interest are the approximate widths of the two wells and of the barrier (or alternatively their frequencies) to account for entropic effects, the barrier height ΔU, and the activity A. The two well-frequencies have been measured experimentally, amounting to $\omega_{\mathrm{L}} = 2\pi \times (73.0 \pm 0.5)$kHz and $\omega_{\mathrm{R}} = 2\pi \times (82 \pm 4)$kHz. The remaining three parameters, i.e. activity, well-separation, and barrier height are tuned to find the best replication of the experimental landscape as presented in the main text (Fig. 3). Initial guesses for the activity and barrier height have been extracted directly from the rate landscape by means of parametric fits. The rate approximation in the regime of high rotational diffusivity directly contains both A as well as ΔU, see Supplementary Equation (15). As the experimental data does not indicate a significant difference between left and right barrier height, we assume them to be equal.

We employ a piecewise-parabolic potential consisting of three segments as the conservative

trapping/barrier force, allowing us to straightforwardly prescribe the well frequencies:

$$U(x) = \begin{cases} \frac{m\omega_{\mathrm{L}}^2}{2}(x - a_{\mathrm{L}})^2 & \text{for } x < b_{\mathrm{L}}, \\ -\frac{m\omega_{\mathrm{B}}^2}{2}x^2 + \Delta U & \text{for } b_{\mathrm{L}} \leq x \leq b_{\mathrm{R}}, \\ \frac{m\omega_{\mathrm{R}}^2}{2}(x - a_{\mathrm{R}})^2 & \text{for } x > b_{\mathrm{R}}. \end{cases} \tag{26}$$

Imposing the aforementioned parameters and additionally demanding continuity of the potential and its first derivative fully determines the location of minima $a_{\mathrm{L}}, a_{\mathrm{R}}$ and connection points between segments $b_{\mathrm{L}}, b_{\mathrm{R}}$. The expressions for $a_{\mathrm{L}}, b_{\mathrm{L}}$ read

$$\begin{aligned} a_{\mathrm{L}} &= -\sqrt{\frac{2\Delta U(\omega_{\mathrm{L}}^2 + \omega_{\mathrm{B}}^2)}{m\omega_{\mathrm{B}}^2\omega_{\mathrm{L}}^2}}, \\ b_{\mathrm{L}} &= \frac{\omega_{\mathrm{L}}^2 a_{\mathrm{L}}}{\omega_{\mathrm{B}}^2 + \omega_{\mathrm{L}}^2}. \end{aligned} \tag{27}$$

The expressions for the right side are obtained by replacing the respective index and changing the sign for a_{R}.

As this approximate one-dimensional potential represents a simplification of the experimental system, we aim to locate a point in parameter space that generates a rate landscape as consistent as possible in selected key aspects. The key aspects considered include the general magnitude of rates and the height, position and width (at half height) of the passive and active turnovers on a logarithmic scale. To this end we apply a bisection-like approach on a discrete grid of parameter values. We consider A, ω_{B}, and ΔU in steps of $0.05\,\mathrm{fN}$, $0.05\omega_{\mathrm{L}}$, and $0.01 k_{\mathrm{B}}T$, respectively. Since the Kramers turnover in the regime of fast rotation is largely independent of A, the respective experimental data can be used to infer the barrier height and frequency/well-separation separately. Leaving the range of potential values more liberty than would be necessary from these initial estimates, we limit the search intervals to $\Delta U/k_{\mathrm{B}}T \in [3.0, 5.0]$ and $\omega_{\mathrm{B}}/\omega_{\mathrm{L}} \in [0.5, 2.0]$. The permissible activity values are restricted by the experimental estimate in conjunction with its symmetric uncertainty. We find a very good agreement between simulation and experiment in terms of turnover heights and widths with the parameter set $A = 9.35\,\mathrm{fN}$, $\omega_{\mathrm{B}} = 0.60\omega_{\mathrm{L}}$ (translating into a minima separation of $0.94\,\mu\mathrm{m}$), and $\Delta U = 4.26 k_{\mathrm{B}}T$.

The position of the calculated Kramers turnover coincides with the experimental result within its resolution and uncertainties as well, leaving the position of the active turnover in terms of D_{R} as the only clear discrepancy despite the ad hoc nature of the potential's functional form. The ratio of the simulated and the experimental position of the turnover on the D_{R}-axis amounts to approximately 2.24, with the simulation placing it at a lower diffusivity. Simulations performed in- and outside the delineated optimization range do not hint at the possibility of situating the two lines of maxima at their targeted positions simultaneously. It appears that the ratio between the active and passive turnover positions remains roughly constant over an extensive regime of parameter values. A possible explanation of this phenomenon lies in both being closely tied to the average transition path time. The Kramers turnover occurs when the particle loses energy on the scale of one $k_{\mathrm{B}}T$ throughout a transition. Similarly, the active turnover stems from the propulsion decorrelating over the duration of a typical transition. Both turnover

locations are thus expected to be inversely proportional to the average transition path time in good approximation. In one dimension it becomes therefore difficult to change the position of one maximum independently from the other. Multidimensional potential landscapes, on the other hand, offer additional freedom by allowing for a multitude of transition paths that differ already in position space. Transitions induced purely by thermal fluctuations as captured and collected in the Kramers phenomenology typically look for paths crossing the lowest point in the barrier as preferred transition channels, even if this point does not lie on a straight line with the minima. On the contrary, activity-induced transition events are likely to follow a more "direct" approach, offsetting an increased barrier height in proportion to the magnitude of A. The different channels associated with active and passive transitions generally translate in distinct transition path times and thus location of the turnover maxima. As the experiment naturally unfolds in a three-dimensional setup, and not all coupling effects between dimensions can be erased in practice, it seems sensible to assume that the lowest energy paths would neither follow a straight line nor perfectly coincide with the active force axis. Any small misalignments of the active force, well- and barrier minima, major axes of the trapping forces in their parabolic approximation etc. could induce this effect.

Finally, it is important to once again underline the consistency of the simulation results with the experimental rates despite the usage of an ad hoc potential. Even the most strongly deviating property, the active turnover's position, only does so by a factor of $c = 2.24$, residing in the same order of magnitude. Given a rotational diffusion axis rescaled by this calibration factor, the obtained replicated landscape becomes quantitatively consistent with the experiment almost everywhere, supporting the models discussed in this document.

SUPPLEMENTARY NOTE 6: ACTIVE NOISE

Statistical properties

In a reduced phase space consisting of only position x and velocity v (but not the angle φ), active propulsion is treated as a source of colored noise with persistence time τ_A. In this section, we derive the most central properties of the active force $n(t) = A\cos(\varphi(t))$ from Equation (1). These also serve as consistency tests for the experimentally implemented noise source.

We start by computing the stationary distribution $p(n)$ of $n(t)$. Noting that φ evolves randomly according to Brownian motion on the interval $[0, 2\pi)$ with periodic boundary conditions, the stationary distribution of φ must be uniform, $p(\varphi) = 1/(2\pi)$. Applying a change of variable from φ to n yields

$$p(n) = \int_0^{2\pi} d\varphi \, \frac{1}{2\pi}\delta(n - A\cos\varphi) = \frac{1}{A\pi|\sin\varphi(n)|} = \frac{1}{\pi\sqrt{A^2 - n^2}}. \tag{28}$$

Next, we direct our attention to the autocorrelation $R_{nn}(t)$ and to the power spectral density $S_{nn}(\omega)$. As the angle evolution represents a Wiener process rescaled by the prefactor $\sqrt{2D_\mathrm{R}}$, increments of the angle throughout a time step Δt are independently distributed according to a Gaussian with variance $2D_\mathrm{R}\Delta t$

$$p(\Delta\varphi = \varphi(t + \Delta t) - \varphi(t)) = \frac{1}{\sqrt{4\pi D_\mathrm{R}\Delta t}} e^{-\frac{\Delta\varphi^2}{4D_\mathrm{R}\Delta t}}. \tag{29}$$

In Supplementary Equation (29), the periodicity of φ has not been taken into account yet. We impose without loss of generality $\Delta t \geq 0$. For any real stochastic process, we recover the negative time interval values from the property $R_{nn}(\Delta t) = R_{nn}(-\Delta t)$. The activity's autocorrelation can be evaluated as

$$R_{nn}(\Delta t) := \langle n(t+\Delta t)n(t)\rangle = A^2 \int_0^{2\pi} d\varphi \int_{-\infty}^{\infty} d\Delta\varphi \ \cos(\varphi)\cos(\varphi+\Delta\varphi)p(\varphi)p(\Delta\varphi). \tag{30}$$

Applying Euler's relation $\cos x = (e^{ix}+e^{-ix})/2$ and

$$\left\langle e^{i\varphi}\right\rangle = 0, \qquad \left\langle e^{i\Delta\varphi}\right\rangle = e^{-D_{\mathrm{R}}\Delta t},$$

leads us to an exact expression for the autocorrelation

$$R_{nn}(\Delta t) = \frac{A^2}{2} e^{-D_{\mathrm{R}}|\Delta t|}. \tag{31}$$

Consequently invoking the Wiener-Khintchin theorem we obtain the power spectral density $S_{nn}(\omega)$ (the shorthand *c.c.* denotes the complex conjugate):

$$S_{nn}(\omega) = \frac{1}{2\pi}\int_{\mathbb{R}} \mathrm{d}t \ R_{nn}(t)e^{-i\omega t} = \frac{A^2}{2\pi}\int_{\mathbb{R}+} \mathrm{d}t \ e^{-(D_{\mathrm{R}}+i\omega)t} + c.c. = \frac{A^2}{\pi}\frac{D_{\mathrm{R}}}{\omega^2+D_{\mathrm{R}}^2}. \tag{32}$$

Experimental Characterization

Figure 2b in the main text depicts a histogram of the active force signal at the output of the FPGA. The output range of the FPGA is $[-1\,\mathrm{V}, 1\,\mathrm{V}]$, meaning that $A = 1\,\mathrm{V}$. The histogram in Fig. 2b agrees very well with Supplementary Equation (28) for $A = 1$. Fig. 2c shows the power spectral density S_{nn} of the active force for three different values of the rotational diffusion D_{R}. We fit S_{nn} with Supplementary Equation (32) and extract the cutoff frequency f_c. The cutoff frequency allows us to calibrate the physical value of the rotational diffusion using $D_{\mathrm{R}} = 2\pi \times f_c$. The value of A measured in newton is estimated from the position response of a particle to a known modulated voltage. The experimental value of A equals $(6.8 \pm 3.4)\,\mathrm{fN}$. The uncertainty in the estimation of the activity is limited by the uncertainty in the calibration factor of the position measurement [4]. Specifically, in order to determine a force in newton, we make use of the mass-dependent transfer function of the particle. An uncertainty up to 50% in the mass of the particle, the main precision bottleneck of the position calibration, translates into the same relative uncertainty for the estimation of the activity. Note, however, that the estimate of A/m does not suffer from such uncertainty.

SUPPLEMENTARY REFERENCES

[1] Sivak, D. A. & Crooks, G. E. Near-equilibrium measurements of nonequilibrium free energy. *Phys. Rev. Lett.* **108**, 150601 (2012).

[2] Dellago, C. & Bolhuis, P. G. Transition path sampling and other advanced simulation techniques for rare events. *Adv. Polym. Sci.* **221**, 167–233 (2009).

[3] Sivak, D. A., Chodera, J. D. & Crooks, G. E. Time step rescaling recovers continuous-time dynamical properties for discrete-time Langevin integration of nonequilibrium systems. *J. Phys. Chem. B* **118**, 6466–6474 (2014).

[4] Hebestreit, E. *et al.* Calibration and energy measurement of optically levitated nanoparticle sensors. *Rev. Sci. Instrum.* **89**, 033111 (2018).
